# A new strategy to map landslides with a generalized convolutional neural network

**DOI:** 10.1038/s41598-021-89015-8

**Published:** 2021-05-06

**Authors:** Nikhil Prakash, Andrea Manconi, Simon Loew

**Affiliations:** Engineering Geology, Department of Earth Sciences, ETH Zurich, 8092 Zurich, Switzerland

**Keywords:** Geomorphology, Natural hazards, Geology

## Abstract

Rapid mapping of event landslides is crucial to identify the areas affected by damages as well as for effective disaster response. Traditionally, such maps are generated with visual interpretation of remote sensing imagery (manned/unmanned airborne systems or spaceborne sensors) and/or using pixel-based and object-based methods exploiting data-intensive machine learning algorithms. Recent works have explored the use of convolutional neural networks (CNN), a deep learning algorithm, for mapping landslides from remote sensing data. These methods follow a standard supervised learning workflow that involves training a model using a landslide inventory covering a relatively small area. The trained model is then used to predict landslides in the surrounding regions. Here, we propose a new strategy, i.e., a progressive CNN training relying on combined inventories to build a generalized model that can be applied directly to a new, unexplored area. We first prove the effectiveness of CNNs by training and validating on event landslides inventories in four regions after earthquakes and/or extreme meteorological events. Next, we use the trained CNNs to map landslides triggered by new events spread across different geographic regions. We found that CNNs trained on a combination of inventories have a better generalization performance, with a bias towards high precision and low recall scores. In our tests, the combined training model achieved the highest (Matthews correlation coefficient) MCC score of 0.69 when mapping landslides in new unseen regions. The mapping was done on images from different optical sensors, resampled to a spatial resolution of 6 m, 10 m, and 30 m. Despite a slightly reduced performance, the main advantage of combined training is to overcome the requirement of a local inventory for training a new deep learning model. This implementation can facilitate automated pipelines providing fast response for the generation of landslide maps in the post-disaster phase. In this study, the study areas were selected from seismically active zones with a high hydrological hazard distribution and vegetation coverage. Hence, future works should also include regions from less vegetated geographic locations.

## Introduction

Landslides are a major concern in regions with mountainous terrain, as they directly impact human lives and infrastructures^1^. Landslide events are often triggered by earthquakes, extreme meteorological occurrences, or anthropogenic activities. A single trigger can induce hundreds of slope failures distributed over a large area^2^. For example, the seismic shaking caused by the 2015 $M_w$ 7.8 Gorkha earthquake affected an area of more than $35{,}000 \; \text{km}^2$ in central Nepal, triggering at least 25,000 landslides^3,4^. Information on the spatial distribution of these landslides in the form of a compiled inventory is important for planning disaster response, understanding landform evolution, identifying hazardous areas, and/or determining risks^3,5^. Such inventories are also essential to forecast the landslide generating potential of similar future events (in terms of magnitude and spatial distribution)^6,7^.

Landslides can be mapped using satellite imagery by identifying characteristic changes in surface features associated with mass wasting. This reduces the dependency on field mapping campaigns and makes the interpretation of remote sensing data the most preferred method for generating inventories of large areas. The increase in the number of Earth observation (EO) satellites and shorter revisit periods have facilitated this approach. It is also possible to fetch the archived pre-event satellite images from the past and compare it with the post-event images. However, mapping is a challenging task as the surface features that help us identify a landslide can vary widely with the data sources, characteristics of trigger, type of movement, geology of the region, and local geomorphology. Hence, this task has been largely dependent on manual or semi-automated methods^7^. Automated methods, based on machine learning algorithms, are being developed to exploit the vast amount of available EO resources^8^. In this direction, the traditional pixel-based and object-based methods have been widely used for mapping landslides^6, 9–11^. Recent advances in deep learning have caused a paradigm shift in computer vision algorithms used to extract information from images^12–15^. They are increasingly being adopted by the geosciences community to find solutions for existing challenges^16^.

Many deep learning implementations to map landslides have been proposed in recent years, which typically use a convolutional neural network (CNN) to learn directly from EO data^17–21^. These implementations have outperformed traditional methods. Ghorbanzadeh et al.^17^ used RapidEye images combined with topographic information derived from the 5 m ALOS digital elevation model (DEM) to train a CNN for detecting landslides. Sameen and Pradhan^18^ added residual connections in their network architecture to map landslides using aerial images with topographic information from a lidar-derived DEM. The residual connections are identity mapping functions that are used to construct alternate skip connections between the convolutional layers. Using residual connections makes it easier for deep neural networks to optimize during the training process^13^. In these standard CNN architectures, a series of convolutional layers/blocks are followed by fully-connected (FC) layers to predict the output class labels. However, a U-Net replaces the FC layers with convolutional decoder blocks to output a segmentation image^14^. Prakash et al.^19^ used a modified U-Net with ResNet34 backbone to perform semantic segmentation of historic/old landslides from topographic features derived from LIDAR data. Different variants of U-Net have also been used in multiple studies to map landslides from optical images in different geographical regions^22–24^. These studies adopt a conventional supervised learning workflow where a model is first trained in a controlled region (also known as the training region) and then reused to generate a landslide map of its surroundings with comparable geo-environmental characteristics. In this approach, even the data source is often not changed during the training and prediction processes. Models trained and validated using this approach cannot be adopted for a fully autonomous pipeline that can rapidly map the new landslides after a post-event image is acquired. However, deep learning architectures are known to generalize relatively well to unseen data and therefore are often used in practice^25,26^. To truly harness the potential of deep learning, we need to change our strategy from what has been used for traditional machine learning approaches.

In this work, we envision a scenario where a new landslide inventory is required immediately after a disaster event that might have triggered multiple landslides over large areas. Machine learning algorithms trained and validated using the existing approaches cannot be adopted for fully autonomous mapping of the new landslides. Such approaches require a local landslide inventory created specifically for the event to train the deep learning models. Depending on the geographic location and availability of resources, this inventory must be generated using manual or semi-automated methods, which is a bottleneck for any rapid mapping application^4^. To the best of our knowledge, no study has yet explored the possibility of creating a generalized deep learning model for mapping landslides beyond the spatial and temporal bounds of its training dataset.

Here, we propose a new combined training strategy to build a CNN-based semantic segmentation model generalized for identifying changes associated with landslide activity using a set of pre- and post-event EO images. For this, we have used a deep network architecture, a large training dataset from multiple sensors and landslide inventories, and strong data-augmentation to make the CNN learn features to map landslides generated by an unseen/future triggering event. Multiple experiments have been designed to validate the proposed deep learning method in different operational scenarios. (I) The first set of experiments explores the potential of the deep learning method. These experiments adopt a conventional approach to train a separate CNN model for each area of interest. (II) The next set of experiments explores the idea of having a common CNN model trained on a combined inventory from multiple study areas. (III) Lastly, the CNNs’ generalization ability is tested by applying them to new regions/triggers that were never encountered in the training process. In addition, because the scale of analysis has always been crucial in landslide mapping algorithms, we also explore how the resolution of inputs to the CNNs influences all three experiments.

In the following, the term ‘landslide event’ or “landslides” will always refer to a group of slope failure phenomena generated by a single trigger, i.e., an earthquake or extreme rainfall events. The term ‘inventory’ will refer to a catalogue of landslides induced by one trigger.

## Study areas and dataset

**Figure 1:**
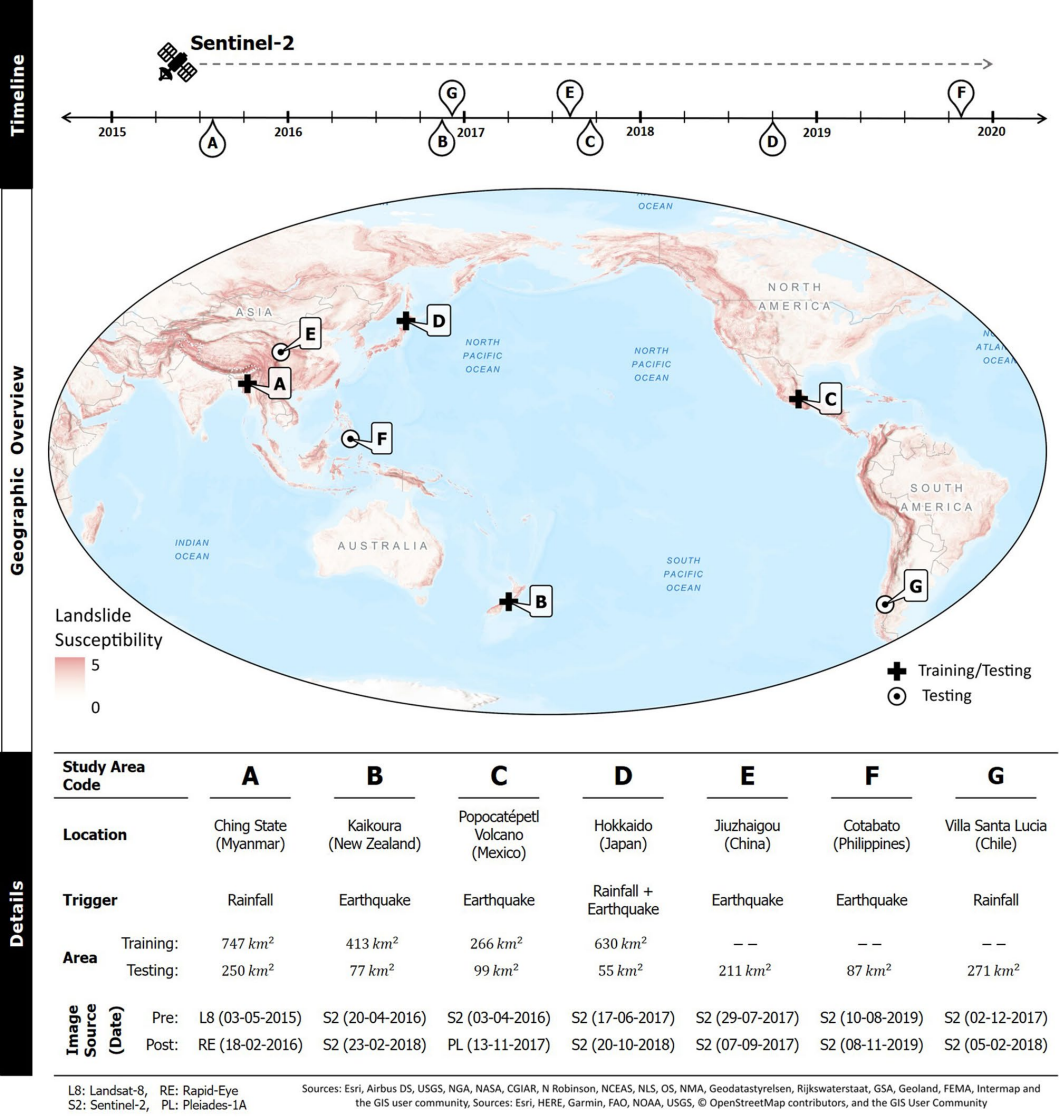
The map shows a geographic overview of the study areas over a background of global landslide susceptibility^51^. They are located around the Pacific Ocean, which is a seismically active zone with a high hydrological hazard distribution^52^. The approximate date of occurrence for the events are marked on a timeline (top). More details on the type of trigger, area of coverage, and optical data used for landslide mapping are presented in the table (bottom). Only study areas A to D will be used for training the CNN models in this work. The magnitude-frequency for all the landslide inventories is shown in Supplementary Figure 1. The map was created using ArcGIS Pro v2.4 (https://www.esri.com/).

We selected a total of seven study areas (identified by letters A to G), which experienced landslide events associated to different triggers. We intentionally chose events after mid-2015 to exploit the data from Sentinel-2 images, which are available for free at a spatial resolution of 10 m. This ensured that the deep learning models generated from this work could be easily adapted for mapping in new areas without being dependent on commercial satellites. Figure 1 shows a geographic overview of the study areas along with additional information on the trigger, spatial extent, and dataset used. Mapping landslides from optical data can be hindered by the presence of clouds and thus can only be done in cloud-free regions. Depending on the local weather pattern, the availability of cloud-free days can be scarce in the area of interest. There could be a delay in the order of months to get an image of the affected region under clear skies. In this work, the satellite image tiles were selected with a low cloud cover without increasing the temporal distance from the triggering event. The topographic information for all the study areas was derived using the ALOS Global Digital Surface Model (AW3D30), which is available at a resolution of 30 m.

### Study area A (Myanmar)

In 2015, the Ching State of Myanmar experienced Cyclone Komen in July, along with unprecedented extreme rainfalls during the monsoon months. The Arakan mountain range covers a significant portion of the Ching state with steep slopes and unstable geologic conditions^27^. This led to widespread flooding and landsliding, which caused extensive damage to infrastructure and loss of life. For this study area, we selected an extent of 990 $\text{km}^2$ in the north of Hakha city. The inventory from an existing work done by Alvioli et al.^28^ was used to train the deep learning models. The authors have used pre- and post-event RapidEye images to map 2131 event landslides, ranging from 162 $\text{m}^2$ to 6.2 $\text{km}^2$. Many large landslides were recorded in this area, where the largest was the Tonzang landslide with a runout length of more than 5 km.

### Study area B (New Zealand)

The South Island of New Zealand experienced a $M_w$ 7.8 earthquake on 14 November 2016, with its epicenter approximately 60 km south-west of Kaikoura. This event triggered more than 10,000 landslides in sparsely populated regions, which fortunately resulted in no reported landslide-related fatalities^29^. However, these landslides caused substantial damage to infrastructure and dammed rivers at multiple locations. We selected a 490 $\text{km}^2$ region between the earthquake epicenter and Kaikoura city as our study area. Multiple works have been done for mapping the landslides triggered by the Kaikoura earthquake using high-resolution images^29,30^. In this work, a new landslide inventory was created to train the deep learning models. A total of 547 landslides were mapped with affected areas ranging from 112 $\text{m}^2$ to 0.5 $\text{km}^2$.

### Study area C (Mexico)

The $M_w$ 7.1 Puebla earthquake, which occurred on 19 September 2017, caused severe damage to life and property in Central Mexico. Coincidentally this earthquake happened exactly 32 years after the devastating Mexico City earthquake on 19 September 1985. Hundreds of shallow landslides were triggered on the flanks of the Popocatepetl volcano, which is located approximately 70 km south-west of the epicenter^31^. The study area selected for this event encloses the Popocatepetl volcano and has a total extent of 365 $\text{km}^2$. In this work, a new landslide inventory was created to train the deep learning models. A total of 754 landslides were mapped with areas ranging from 216 $\text{m}^2$ to 0.7 $\text{km}^2$.

### Study area D (Japan)

The $M_w$ 6.6 earthquake occurred in Hokkaido, Japan, on 6 September 2018. This happened only a few days after this Northern island of Japan witnessed extensive rainfall due to Typhon Jebi. The seismic shaking triggered an unusually high density (326 per $\text{km}^2$) of co-seismic landsliding, which was observed in an area of 700 $\text{km}^2$^32^. We considered this region as our study area, and the landslide inventory was used from an earlier published work^32^. The authors manually mapped the co-seismic landslides using a set of PlanetScope satellite images acquired on 11 September 2018. They were able to reject the non-coseismic landslides by manual cross-checking with pre-earthquake images collected on 22 March and 3 August 2018. The inventory catalogs 7837 landslides with areas ranging from 74 $\text{m}^2$ to 0.085 $\text{km}^2$, which were used to train the deep learning models.

### Study area E (China)

The Sichuan Province in China was struck by the $M_w$ 6.5 Jiuzhaigou Earthquake on 8 August 2017. This induced many landslides in the form of small scale rockfalls and rock/debris slides^33^. For this event, we selected a 200 $\text{km}^2$ region as our study area. In this work, a new landslide inventory was created for this study area for testing the deep learning models trained on other study areas. A total of 227 landslides were mapped with affected areas ranging from 147 $\text{m}^2$ to 0.1 $\text{km}^2$.

### Study area F (Philippines)

The province of Cotabato in the Philippines was struck by a swarm of earthquakes in October 2019. Many of these events had a magnitude above $M_w$ 5, with the highest magnitude reaching $M_w$ 6.6. These earthquake events triggered multiple landslides in two clusters. This is the most recent event on our list, and it was difficult to find cloud-free images in Sentinel-2 and Google Earth Pro archives. For this study, we carefully selected an 87 $\text{km}^2$ study area to manually map the landslides in the southern cluster. This inventory has 309 mapped landslides with affected areas ranging from 594 $\text{m}^2$ to 0.19 $\text{km}^2$ and will only be used for testing the deep learning models trained on other areas.

### Study area G (Chile)

The southern Palena province of Los Lagos Region in Chile experienced torrential rainfall on 15th and 16th of December 2017. This triggered a rockslide in the headwaters of Burrito River, which was followed by large debris and mudflows that traveled for more than 8 km and caused heavy destruction in Villa Santa Lucia^34^. Unlike the previous study areas with hundreds of landslides, this study area in Chile has just one large landslide, which impacted a total area of 7.73 $\text{km}^2$. The deep learning models trained on other study areas will be used to map the area affected by the Villa Santa Lucia landslide.

## Results

We conducted experiments to train multiple CNNs on inventories from study areas A to D. For every training scenario, we explored the influence of the base resolution on mapping performance. From here on, we follow a naming convention to unequivocally identify an experiment, namely:

$$\begin{aligned}&Trained \; \; CNN \; \rightarrow \; {M_{R}^{SA}} \\&where, \; SA \in \{A,\;B,\;C,\;D,\;ALL\} \\&and, \; R \in \{6\;m,\;10\;m,\;30\;m\} \end{aligned}$$

Here, the super-script *SA* represents the Study Area used for training the CNN. When $SA = ALL$, the CNN was trained on the combined inventories of study areas A to D. Similarly, the sub-script *R* represents the base Resolution used for resampling the inputs to the CNN. For example, the symbol $M_{10}^{A}$ represents a CNN trained on landslide inventory from study area A at a base resolution of 10 m.

### Performance evaluation of conventional learning approach

**Figure 2:**
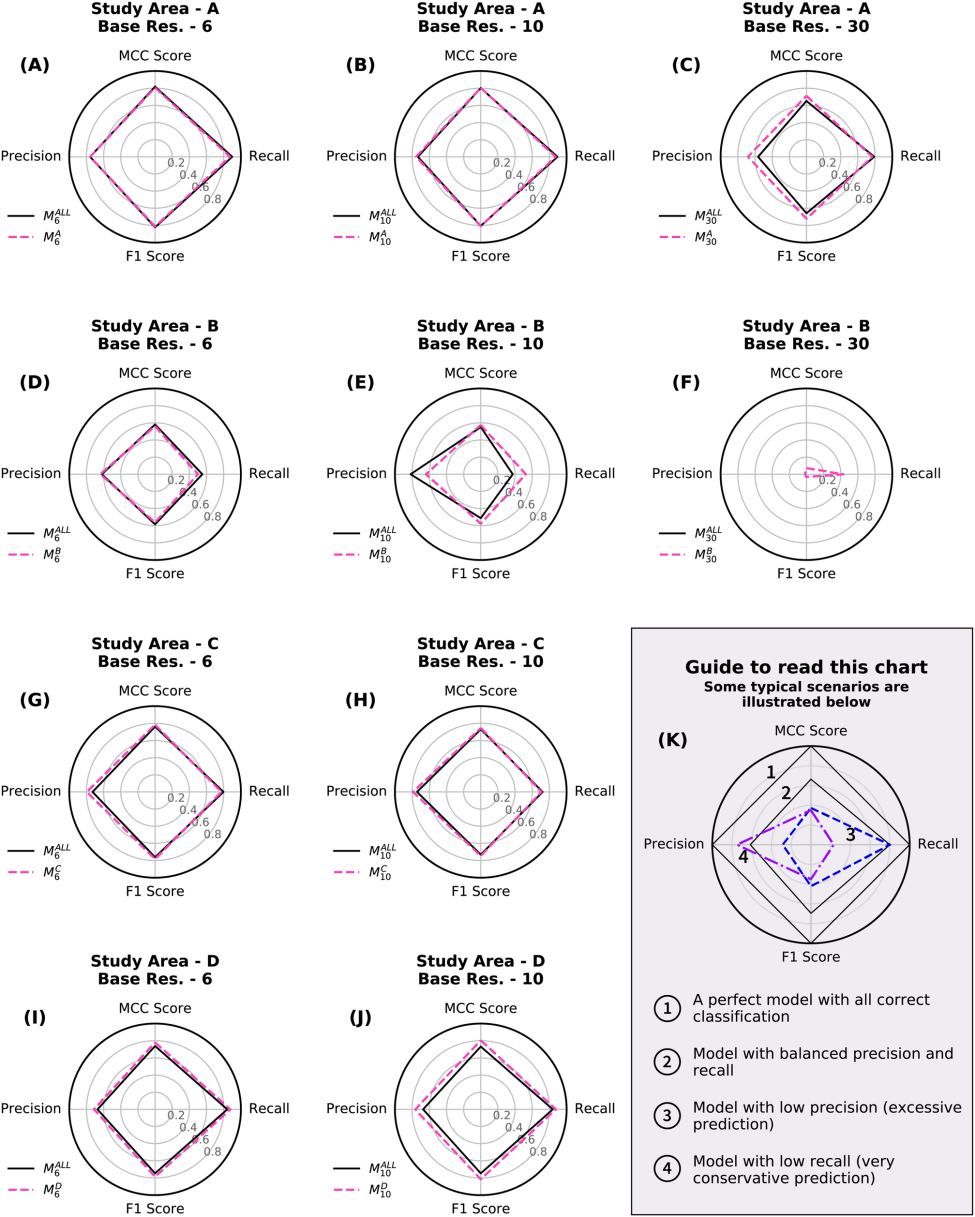
The spider plots (**A–J**) show the performance of CNNs trained with conventional learning (dashed line in pink) and combined learning (solid line in black) approaches. Every row has models evaluated for a specific study area at different base resolutions. The precision and recall scores are plotted on the X-axis, and the MCC and F1 scores are plotted on the Y-axis. (**K**) illustrates the shape expected from a good model which can be differentiated from models with lower precision or recall scores. The envelope of a better model will have a higher MCC/F1 Score and will enclose the plot from a weaker model. (Res. = Resolution).

We trained ten CNN models on each inventory from study areas A to D at different base resolutions. In general, the CNNs were able to learn the features required to map landslides in their respective testing regions. The dashed pink lines in Fig. 2 represent the CNNs trained with a conventional learning approach. The best mapping performance was observed for $M_{6}^{A}$, which got a Matthews Correlation Coefficient (MCC) score of 0.806. The models showed the weakest performance in study area B, with the best MCC score of only 0.574 (by $M_{10}^{B}$). The best performance of study areas A, C, and D was observed while mapping at a base resolution of $6 \; \text{m}$. On the other hand, the best performance of study area B was observed at a base resolution of $10 \; \text{m}$. The CNN takes a tile of $224 \times 224$ pixels as input, which corresponds to a footprint of $1.34 \times 1.34\; \text{km}$ at a base resolution of $6 \; \text{m}$. But when training is done at a base resolution of 30 m, the footprint on the ground increases to $6.72 \times 6.72\; \text{km}$. The testing regions identified in study areas C and D were smaller than $6.72\; \text{km}$ in at least one dimension (easting or northing). As a result, for these two study areas, we were unable to use the CNNs for mapping at a base resolution of 30 m.

### Performance evaluation of combined learning approach

**Figure 3:**
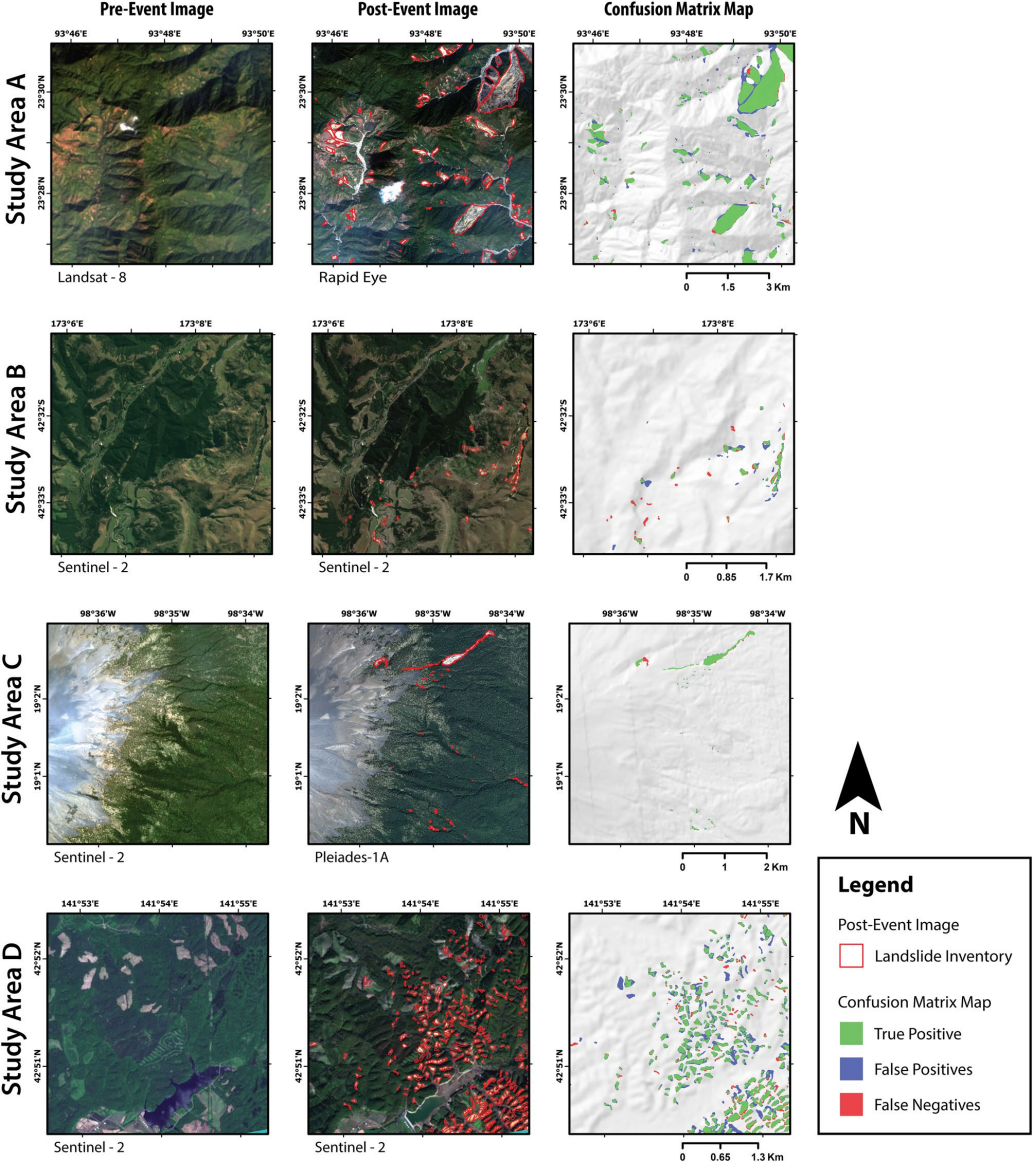
Result of applying the trained CNN model ($M_{6}^{ALL}$) on the testing region of study area “A” to “D”. Every row shows a fixed view from one study area with pre-event image (left), post-event image with polygons from landslide inventory (middle), and output from CNN as a confusion matrix map (right). The majority resampling method was used to visualize the confusion matrix map. The cloud and snow-cover masks have not been shown for clarity, but the affected region was not used in the training and prediction process. The maps were created using ArcGIS Pro v2.4 (https://www.esri.com/).

We trained three CNN models on combined inventories from study areas A to D at different base resolutions. The CNNs trained on combined inventories performed similarly to those trained with a conventional approach (Fig. 2). Mapping landslides with $M_{6}^{ALL}$ in study area A resulted in the best reproduction of the ground truth, with an MCC score of 0.818. Even the combined models showed the weakest performance scores in study area B, where $M_{6}^{ALL}$ produced a maximum MCC score of 0.58 across all the base resolutions. $M_{30}^{ALL}$ failed map anything in this study area. In Fig. 3, we show an example of the confusion matrix map generated by $M_{6}^{ALL}$ for study area A to D to show the true positive (TP), false positive (FP), and false negative (FN) predictions. The true negative (TN) predictions, which are the regions correctly identified as “not-landslide” by the CNNs, are not shown on the map as a separate label.

### Generalization performance

**Figure 4:**
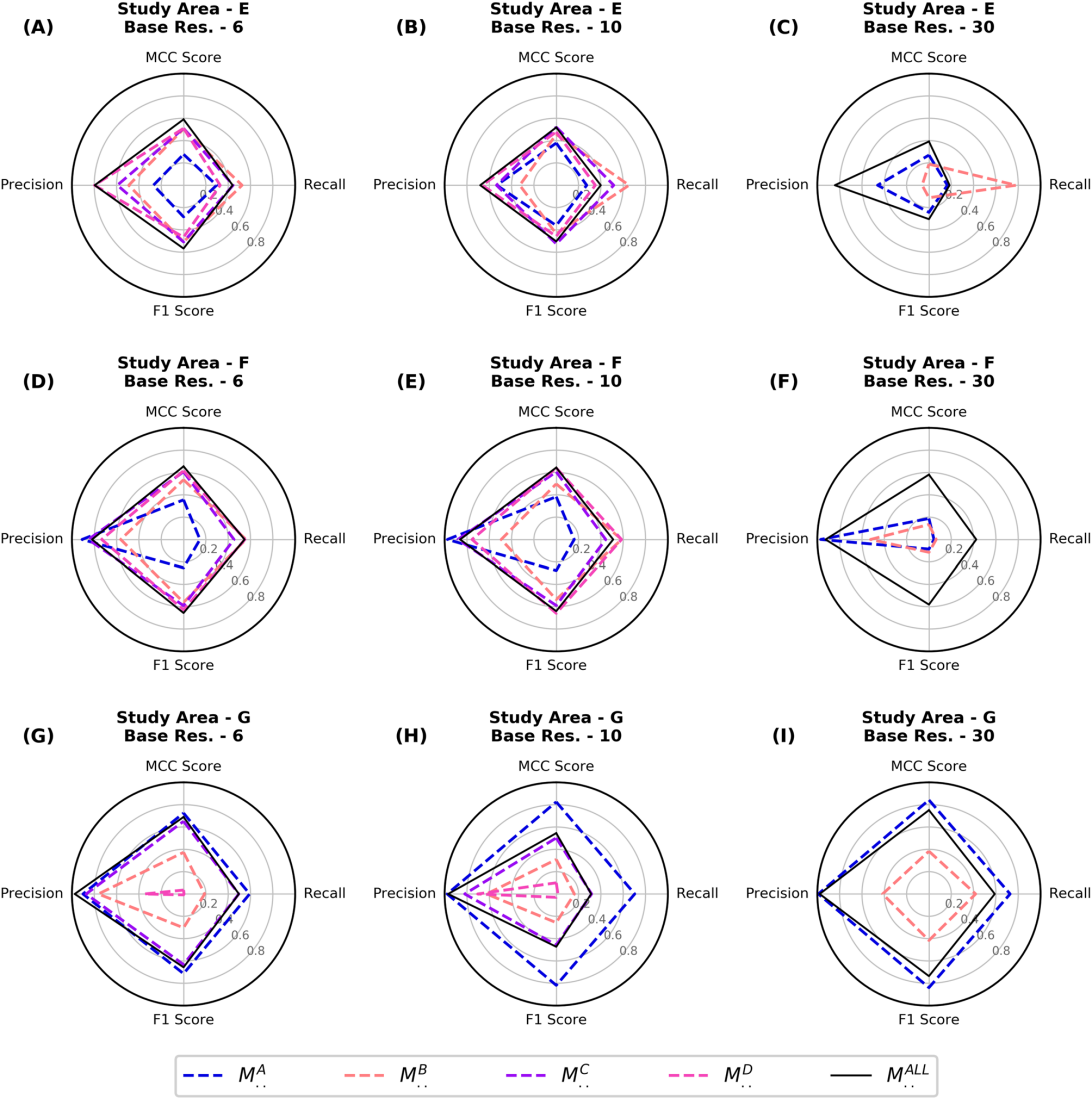
The plots (**A–I**) compare the generalization of conventional learning and combined learning models. Every row evaluates landslide mapping performance for unseen events in the study area “E” to “G”. The guide to read this plot is illustrated in  Fig. 2 (**K**). (Res. = Resolution).

**Figure 5:**
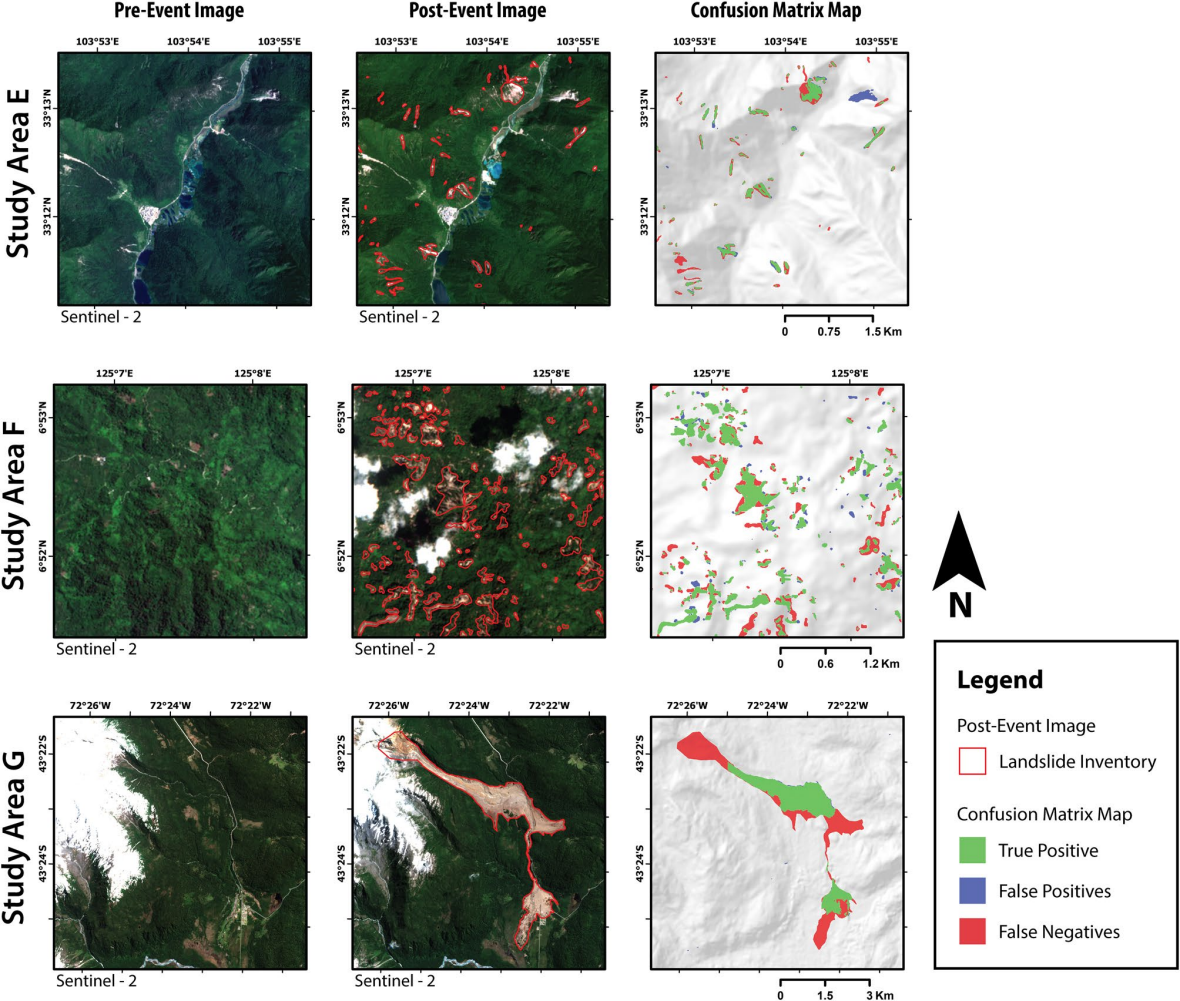
Result of applying the trained CNN model ($M_{6}^{ALL}$) on study area “E” to “G”. These three study areas were not used for training any CNN model; hence they were completely used as the testing region. The results highlight the generalizing ability of $M_{6}^{ALL}$ to map event landslides. Every row shows a fixed view from one study area with pre-event image (left), post-event image with polygons from landslide inventory (middle), and output from CNN as a confusion matrix map (right). The majority resampling method was used to visualize the confusion matrix map. The cloud and snow-cover masks have not been shown for clarity, but the affected region was not used in the training and prediction process. The maps were created using ArcGIS Pro v2.4 (https://www.esri.com/).

To test the generalization performance, the CNNs trained in the above experiments were used to map landslides in a new geo-environmental setting, i.e., without using a local training inventory. Figure 4 compares the performance of all the trained CNNs for mapping landslides in study area E to G. The model $M_{6}^{ALL}$ showed the best performance scores for study areas E and F, with an MCC score of 0.59 and 0.65 respectively. In study area G, the best performance was observed by $M_{30}^{A}$ with an MCC score of 0.842, which was closely followed by $M_{10}^{A}$. We observed the mapping of the CNNs on unseen regions is biased towards high precision with relatively low recall. This means that the area identified as a landslide can be trusted with high confidence, but the inventory is not complete as it also misses out on many landslides. For study area G, the highest recorded precision score was 0.991 and while the recorded highest recall scores were only 0.727 (both for $M_{30}^{A}$). Figure 5 shows an example of the confusion matrix map generated by $M_{..}^{ALL}$ for study area E to G.

## Discussions

CNNs are widely used for automatic interpretation of satellite images to retrieve valuable information. The proposed method performed well for mapping event landslides in their corresponding testing regions. Figure 3K is a guide for interpreting the behavior of the CNNs, which have been visualized on the spider plot. A model with a perfect correspondence to the ground truth will have a value of 1 for MCC, F1, precision, and recall scores. However, it is difficult to accurately delineate the precise boundary of most landslides in the real world. It is common for experts to disagree and have conflicting interpretations of the spatial extent of landslides present in any specific area^35^. We do not expect the landslide map generated by a CNN to be exactly equal to the ground truth inventory. A good performing CNN is indicated by a high MCC score with balanced precision and recall scores. A CNN biased towards low recall and high precision scores will be very conservative in mapping and overlook many landslides affected areas. On the other hand, a CNN with high recall and low precision scores will be very aggressive in mapping by overpredicting many stable slopes as landslides. A model biased towards higher recall values would be generally preferred for an inventory mapping done for risk assessment and disaster management.

In this work, we continuously tracked the consequence of changing the resolution of the input data. Any changes to the base resolution will directly influence the extent of spatial context observed by the CNN in one pass. A tile with a base resolution of $6 \; \text{m}$ corresponds to a ground footprint of $1.34 \times 1.34 \; \text{km} \; (1.8 \; \text{km}^2)$, whereas a tile with a base resolution of $30 \; \text{m}$ will correspond to a ground area of $6.72 \times 6.72 \; \text{km} \; (45.1 \; \text{km}^2)$. Thus, the tile with a smaller base resolution might not see the full extent of a large landslide. This can justify the performance of CNNs tested at a base resolution of $30 \; \text{m}$ in study areas A and G, as both have large landslides triggered by extreme rainfall. However, a tile with a higher base resolution can distinguish finer surface features, essential for identifying landslide affected areas.

In our study, we found the conventional and combined learning models to have a small bias towards higher recall scores when tested on study areas A to D (Fig. 3A–J). The best MCC scores for these study areas were above 0.7, except for study area B. The low scores were caused by many small landslides in the testing region of study area B, which were completely missed by the CNN. All the models in the training study areas showed the weakest performance at a base resolution of $30 \; \text{m}$. We also observe that CNNs trained at a base resolution of $6 \; \text{m}$ always achieve a higher MCC score than similar models at a base resolution of $10 \; \text{m}$. The performance gain between the two base resolutions is not very significant, with improvements ranging from a margin of 0.003 to 0.03. Here, $M_{10}^{A}$ (MCC score: 0.560) is an exception which was outperformed by $M_{10}^{B}$ (MCC score: 0.574). We have used data from high-resolution satellites for the post-event image in study areas A and C (Rapid-eye and Pleiades, respectively). This was done for two reasons: (i) these images from the commercial satellites was available to us, and (ii) we were able to test our method on other medium resolution images apart from Sentinel-2. This higher resolution of inputs could partially explain the marginally higher MCC scores for models trained at a base resolution of $6 \; \text{m}$ in study areas A and C. However, the same advantage is not applicable for study area D, which also shows a similar trend in MCC scores while using Sentinel-2 images. Using images sampled at a higher resolution of $6 \; \text{m}$, we generate more tiles for the same extent of a study area, thereby virtually increasing the number of images available for training. A similar practice of upsampling images to achieve better results exists in other satellite image processing applications like image co-registration^36^.

Unlike what we observe in study areas A to D, we notice that the combined learning method has an advantage in mapping landslides in a new area (Fig. 5). For study areas E and F, the combined learning CNNs consistently performed better than their conventional learning counterparts, with the best results coming from $M_{6}^{ALL}$. The landslides in these two study areas are triggered by earthquakes. This might also explain why conventional learning CNNs’ performance in study areas B to D (also earthquake-triggered) is also not very low. However, the same models showed a poor performance while mapping the rainfall triggered Santa Lucia landslide in study area G. $M_{10}^{A}$ and $M_{30}^{A}$ had the best performance while mapping this large landslide, closely followed by the combined learning model $M_{30}^{ALL}$. An explanation for the poor performance of $M_{..}^{ALL}$ in study area G can be explained by the under-representation of rainfall triggered landslide inventories in the training data.

**Figure 6:**
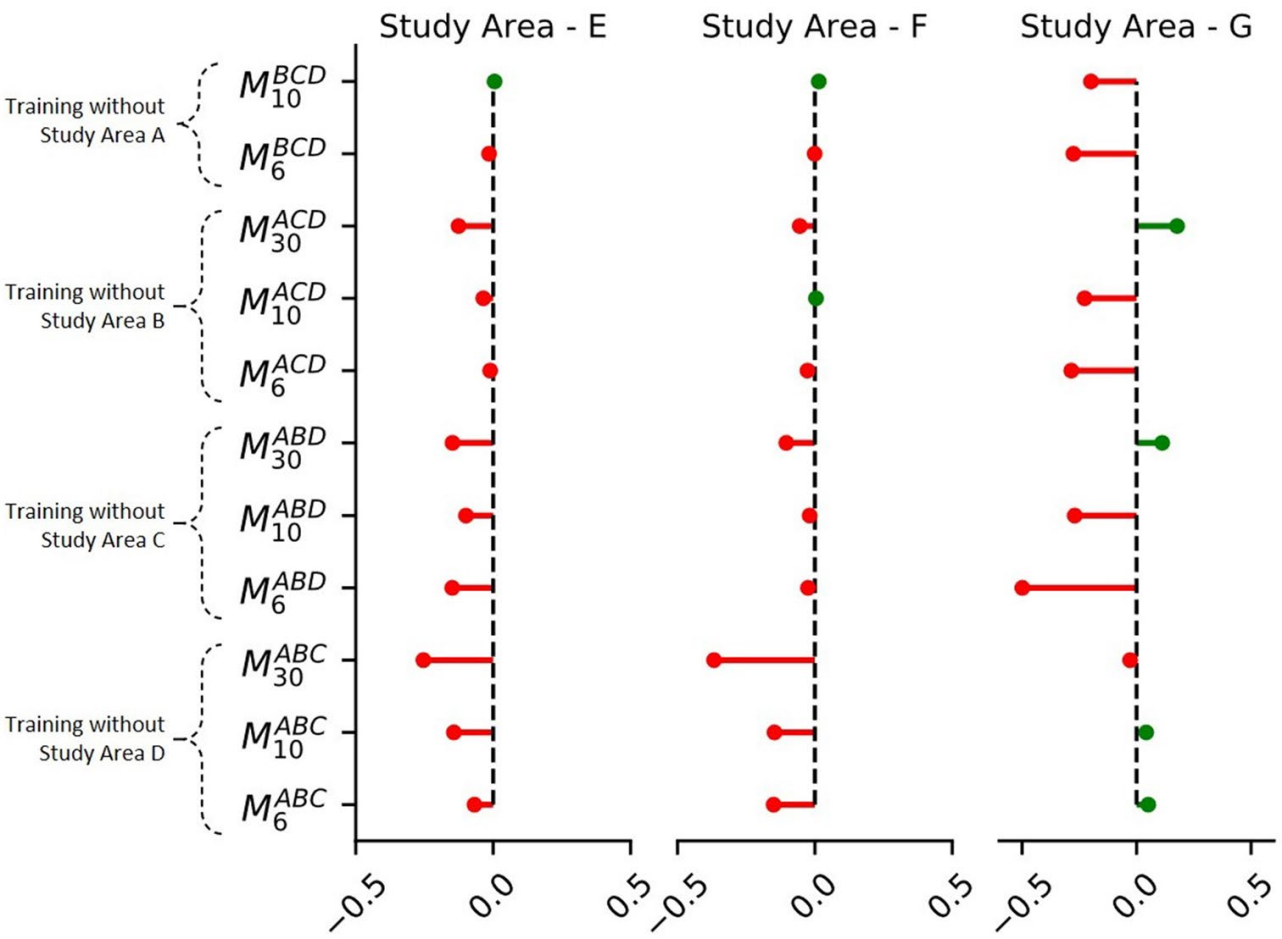
Jackknife’s approach for training new CNNs on combined landslide inventories. Here, we rule out the possibility of a bad inventory bringing down the performance of the combined learning approach. The values in X-axis are the difference of MCC score of the new model with the MCC score of $M_{6}^{ALL}$. A red line shows a decrease in MCC score, and a green line shows an increase in MCC score. An example of nomenclature used here: the symbol $M_{6}^{ABC}$ represents a new combined learning model which has been trained on study area A, B, and C at a base resolution of 10 m.

We found that the CNNs performance on new areas was biased towards higher precision scores. This is similar to observations from other deep learning work done in the area of domain generalization and multidomain learning^37^. To rule out any negative contributions from any of the four training inventories used by the combined learning models, we adopted a jackknife approach by conducting more experiments to train CNNs from only three study areas, while systematically leaving out one each time. Figure 6 shows the changes in MCC scores for these new CNNs as compared to the scores for $M_{6}^{ALL}$. We found that training without rainfall triggered inventory from study area A improves the MCC score in earthquake-affected study areas E and F. But, it also decreases the MCC score for the rainfall-affected study area G. No single inventory removal contributed to an overall improvement in the performance of the combined model. Thus, future efforts should instead be focused on expanding the training dataset by progressively adding new inventories from more landslide events.

The FP and FN detections in the confusion matrix maps are often observed on the boundaries of the ground truth inventories, or due to amalgamation of closely located landslides (Figs. 2 and 4). The FP detections should not be overlooked as they could also point to a new landslide that is missing in the ground truth inventory. As discussed in the above paragraph, the performance of CNNs on study area E to G is biased towards high precision and low recall scores. This can be observed in the confusion matrix maps of Fig. 4, where significant FN detections completely miss out on many landslides. This does not necessarily mean a bad result, as we are still correctly mapping many landslides with high precision without specifically training a new CNN on that particular area. To understand the current situation from a recent event, we can look at the timeline of image acquisition and mapping effort compiled by William et al.^4^ after $M_w$ 7.8 Gorkha earthquake of 25th April 2015. The first two maps were created after one week, which identified less than 500 landslides as point features. It took many weeks to generate a more detailed inventory with 5600 landslides delineated as polylines. For the same disaster, the number of landslides identified grew by a factor of five after a few years of mapping efforts^3^. An algorithm that can rapidly generate a first cut map of areas affected by landsliding after a disaster would be valuable for planing any relief operations. Once more time is available, the affected area can be remapped with the conventional approach using the trained CNN as a pre-trained model for an updated landslide map with higher accuracy.

However, this method does not work if the pre- or post-event optical image has been affected by cloud or snow. The pre-event image used in study area G was covered in snow, resulting in an FN detection in the head scarp region of the Santa Lucia landslide (Fig. 4). This could be a serious problem for some cases as it might take many days for the clouds to clear the sky. Methods working on spaceborne Synthetic Aperture Radar data can provide information even through cloud cover and should be used for such situations^38,39^. All the study areas selected for this study had had high coverage of vegetation. In future works, landslides and EO data from less vegetated geographic locations should be included in the training and testing datasets. Deep learning models are very good at learning from the training dataset. The algorithm also learns the biases of the expert who is mapping landslides for the training process. Hence, high quality of correctness should be ensured for the landslide inventory used for training the model. Using a large dataset of many inventories generated by multiple experts is expected to decrease this problem.

## Conclusions

Automated mapping of landslides is a challenging task. Experts commonly use a set of pre- and post-event images to delineate landslides using manual or semi-automated methods. This study proposed a new strategy for training a deep learning model to map event landslides from medium resolution EO data. Seven post-2015 triggers in different tectonic settings were selected for training and testing the CNN models. New landslide inventories were prepared for four triggers for which public records were not available or were unsuitable for this work. The CNNs were trained separately on four inventories using a conventional supervised learning approach. The performance scores of the trained models were evaluated by applying them to the hold-out regions of their respective study areas. However, a CNN trained using a conventional approach is not expected to map landslides induced by a different trigger from another geographic location.

The lack of training data is a common problem in the effective deployment of a data-driven model for a near real-time landslide mapping task. This study addresses the issue by testing the generalization performance of CNNs to map landslides induced by three triggers that were not seen by the CNN during the training process. Results show that a CNN trained on a combination of landslide inventories from multiple triggers has a better generalization than a similar CNN trained with a conventional approach. We also observed that mapping the landslides induced by new and unseen triggers is biased towards high precision and low recall scores. However, this is not an issue as such CNNs can be used on future triggers in new geo-environmental settings without a re-training step. We also provide access to a pre-trained model and a Jupyter Notebook script, making it convenient to map an event landslide inventory in a new area of interest.

## Methods

In this study, a modified U-Net architecture was used for the automatic semantic segmentation of landslide events. We train the CNN models on landslide inventories from study areas A to D. Hence, the extent of these four study areas is further sub-divided into non-overlapping training and testing regions. The holdout testing region is used for evaluating the performance of the trained models. On the other hand, we do not use study areas E to G in the training process. The full extent of these study areas is used as a testing area for evaluating the trained models’ generalizing ability. The next sub-sections give a detailed description of the procedure we followed to map and evaluate the landslide inventory.

### Visual interpretation of landslides

The landslide inventories are prepared by visually identifying regions affected by the landslide event. A set of pre- and post-event true color composites of Sentinel-2 images along with corresponding AW3D30 digital surface model were used to delineate landslides as a vector polygon. However, as an exception, we used high-resolution Pleiades-1A image acquired on 13 November 2017 for mapping landslides triggered by the Puebla earthquake (study area C).

Mapping landslides from medium resolution satellite images is a challenging task. The optical images were draped over a 3D terrain model to get a better visualization of the topography during the mapping process. High-resolution multi-temporal images available in Google Earth Pro software package were used for identifying very small landslides and doubtful cases. We often noticed minor registration issues when comparing data from multiple sources. As a result, a mapped landslide polygon from one image source appeared with a minor shift on a different image source, especially on some high-resolution Google Earth images. To keep a consistent mapping scheme, the final landslide polygons were created to mark the landslide extent visible on post-event images mentioned in Fig. 1.

It should be noted that the inventory of event-triggered landslides was generated for a limited scope of training a CNN for a segmentation task and lacks any information on the type of movement, volume of the landslide, or its forming material.

### Preprocessing and data augmentation

**Figure 7:**
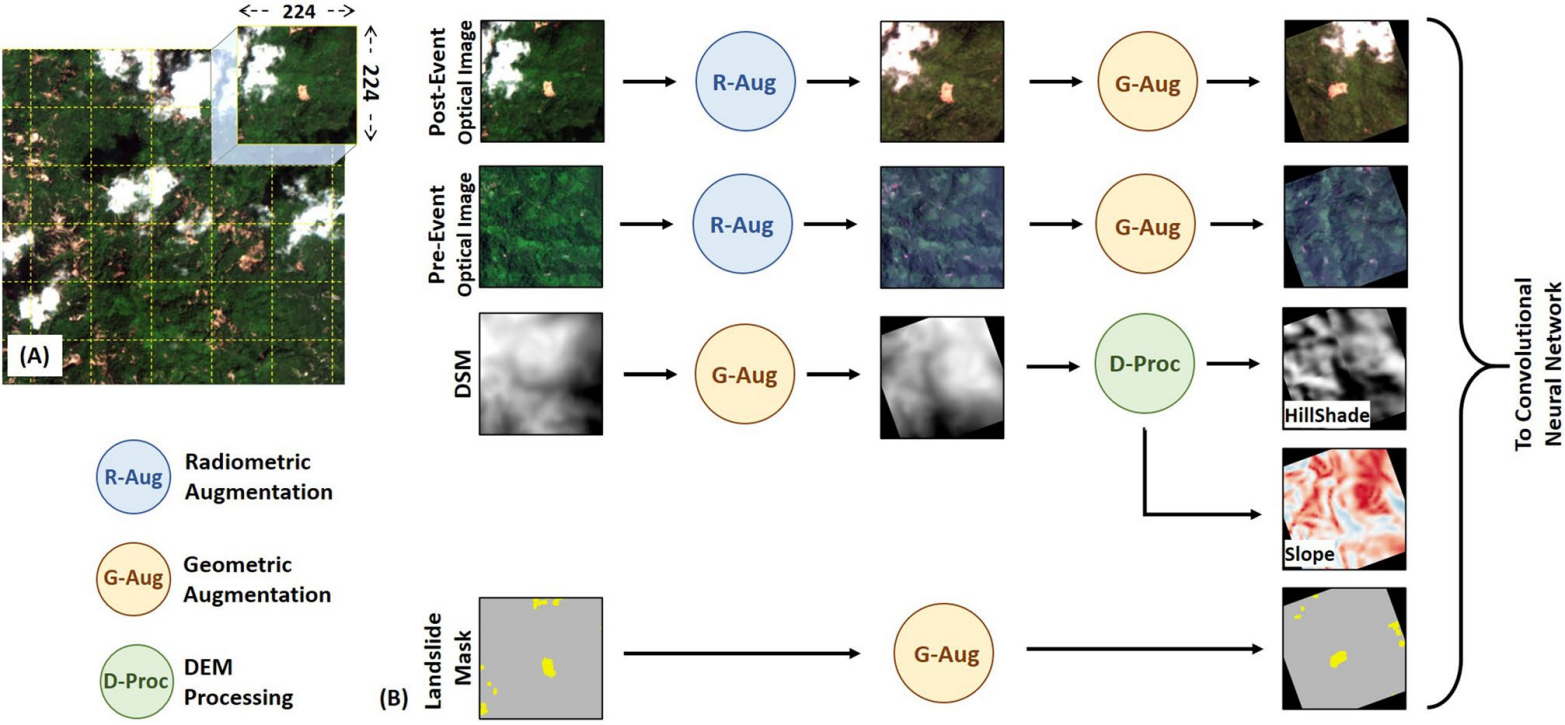
Pre-processing workflow adopted for training of CNN. (**A**) Systematic tiling of input images with 50% overlap. In this study , a tile size of $224 \times 224$ was used as an input to the CNN. The tile size in display is for representation purposes and does not scale to the actual base resolution used in training the CNN. (**B**) Steps involved in the data-augmentation of input images with their corresponding landslide mask.

The first step of preprocessing involved masking out the clouds, shadows, and snow present in the pre- and post-optical images. This mask was created manually for this study, but any automated algorithm can be used for this task^40,41^. All the input data sources were available at different resolutions and had to be resampled to a common base resolution. The dimensions of EO images are generally very large and cannot be used directly as an input to a CNN. To overcome this problem, it has been a common practice to divide the image into smaller and manageable image patches called tiles. In this study, we systematically extracted tiles of $224 \times 224$ pixels from the set of input images (Fig. 7A). While extracting the tiles for the training process, an overlap factor of 50% was used to increase the number of samples available for training the network. The corresponding binary landslide masks were generated by rasterizing the available inventory at the desired base resolution.

The training of CNN requires a large number of labeled images. Also, a typical CNN has millions of trainable parameters, making it very efficient in learning the perfect parameters to over-fit the training data^42^. This limits the generalization ability of the trained model. Data augmentation is a commonly adopted practice in deep-learning to artificially increase the sample count of the training dataset by applying valid random transformations. It also increases the diversity of the training dataset, which helps in regularizing the trained model to improve generalization^42,43^. We applied strong augmentations to the set of input images and landslide masks (Fig. 7B). These augmentations can be broadly categorized into radiometric augmentation (R-Aug) and geometric augmentation (G-Aug). R-Aug includes transformation functions to make random changes in the appearance of images, which include changes in colors, blurring/sharpening, and addition of noise. On the other hand, G-Aug includes transformation functions to alter the geometry of images with random flipping and affine transformations. The state of randomness in R-Aug is different when it is applied to a set of pre- and post-optical images. But the state of randomness in G-Aug is kept constant when it is applied to a set of input images and its corresponding landslide mask. This makes intuitive sense as the pre- and post-images can have some differences in noise and colors. However, if one image from a set is perturbed with geometric transformations like a rotation of +20°, all the other images must be rotated by the same amount to have a coherent set of images. Finally, hillshade and slope maps were generated after G-Aug has been applied to the DEM. The topographic information will help in reducing FP detection in flat regions. The stack of these augmented topographic and optical images paired with the corresponding augmented landslide mask is used to train the CNN.

There are many earth observation satellites in operation. It is possible to observe a landslide event using multiple combinations of pre- and post-event data by varying the source of satellites image and their date of acquisition. In the conventional approach adopted in previous studies, the set of data used during prediction and training is the same. However, a generalized CNN is expected to map landslides from any optical data source. Therefore, it is also possible to increase the number of images available for training by adding more combinations of pre- and post-event images. For training the combined learning models in study areas A and C, we supplement the high-resolution dataset with an extra set of Sentinel-2 image pairs.

### Convolutional neural network

**Figure 8:**
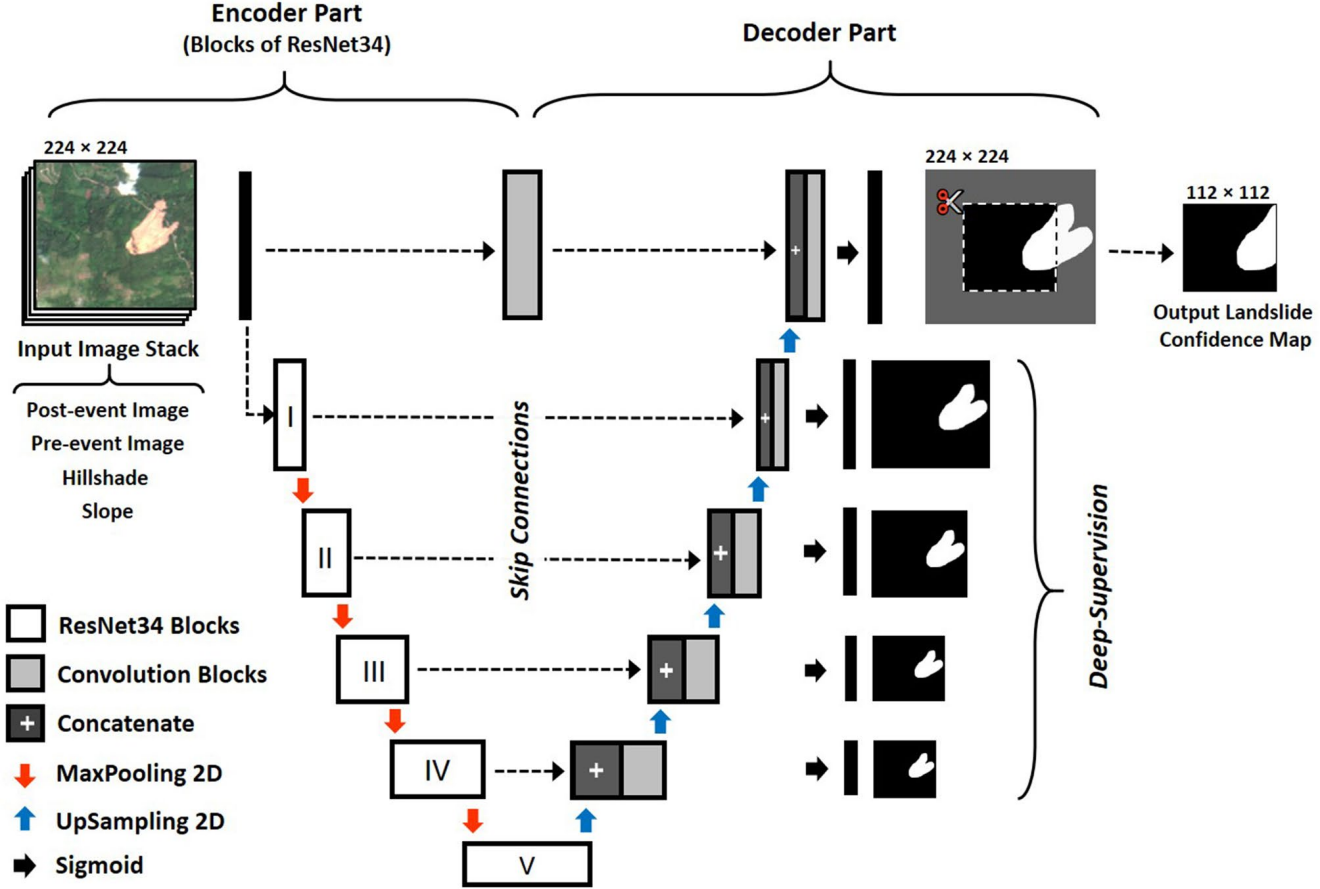
An overview of proposed U-Net architecture with residual blocks and deep-supervision. The complete description of all the layers in the network is available in Supplementary Figure 2.

U-Net has been introduced by Ronneberger et al.^14^ for segmentation of biomedical images and has been adopted for many different applications^19,44^. A U-Net is a fully-convolutional CNN that consists of a downsampling part that acts as an encoder. Skip connections from the encoder part to the decoder part help the CNN recover its full spatial resolution^45^. In this work, we use a modified U-Net, which was made deeper by replacing the convolutional blocks with blocks of residual network with identity mappings (Fig. 8). These residual networks enable the training of deeper models without degrading the network performance^13^. Dropout layers and L2 regularization were used to increase the CNN’s generalization ability. In addition, the U-Net was also deeply supervised at all the blocks in the upsampling part^15^.

In the proposed architecture, ReLU was used as the activation function after every convolution operation. The final prediction layer is generated after a $1 \times 1$ convolution with sigmoid activation. This generates an output image with confidence values ranging from 0 to 1, which is thresholded at 0.5 to get a binary segmentation mask. During prediction, an overlap-tile strategy was applied by selecting the center $112\times 112$ pixels of the output image for generating a seamless segmentation map of very large images^14,19^.

### Training process

The entire process is implemented in Python using GDAL^46^ for GIS processing, Keras^47^ and TensorFlow^48^ for machine learning. Before the start of training, a small section of the training area was kept aside for validation.

We used a combination of focal Tversky index (FTI) and binary cross-entropy (BCE) as a loss function during the training process^19,49^. The total loss $({\mathscr {L}})$ is a weighted sum of the loss from the deeply supervised layers $({\mathscr {L}}^i, \; i = 1,2,3,4)$ and the final output layer $({\mathscr {L}}^0)$.

$$\begin{aligned} {\mathscr {L}} = \sum _{i=0}^{4} w_i {\mathscr {L}}^i \end{aligned}$$

The value of hyper-parameter $w_0 = 5$ and $w_{1-4} = 1$. Also, ${\mathscr {L}} ^i$ is given by:

$$\begin{aligned} {\mathscr {L}}^i = 0.2 \times {\mathscr {L}}^i_{BCE} + 0.8 \times {\mathscr {L}}^i_{FTI} \end{aligned}$$

Adam optimizer was used to minimize the total loss $({\mathscr {L}})$ with a learning rate of $10^{-4}$. After every epoch of training, the validation loss (total loss in the held-out validation set) was evaluated. The learning rate was decreased by 0.1 if the validation loss plateaued or started rising for three continuous epochs. The training process was stopped if there was no further drop in validation loss was recorded for ten continuous epochs. The model with the lowest validation loss during the training process was used to map landslides in testing regions.

### Performance evaluation

CNN’s performance was evaluated by applying the trained model to the holdout testing areas. The landslide inventories used in this work served as the ground truth labels. The binary map of predicted landslides was compared with the ground truth to generate a map of confusion matrix values (for e.g., Figs. 2 and 4), i.e., TP, FP, FN, and TN values. Here, TP represents the correctly predicted landslides, and TN represents correctly identified regions with no landslide activity. The FP represents the landslides predicted by the CNN, which is missing in the ground truth. Similarly, the FN represents the landslides that the CNN has missed. In the next steps, these confusion matrix values were used to calculate few commonly used metrics for the statistical analysis of the binary classification. Ideally, in machine learning, the accuracy score is considered a reliable metric for evaluating a trained model’s performance. However, landslide classification is a highly unbalanced learning task where the spatial extent of the landslide affected area is much smaller than the stable areas. As a result, the accuracy score becomes unreliable due to very high true negative values. This study reports the F1-Score, MCC score, precision, and recall values plotted in a spider chart (Table 1). We consider the MCC score to be the most suitable metric for comparing and ranking the CNN models tested in this work^50^. Understanding an MCC score is also intuitive for a binary classifier, with 0 indicating a model with no correlation (random guesses) and 1 indicating a perfect correlation (all correct guesses).

**Table 1:** Derivation of performance evaluation metrics from confusion matrix values.

Metric	Formula
F1-score	$\frac{2TP}{2TP+FP+FN}$
MCC	$\frac{TP \times TN - FP \times FN}{\sqrt{(TP + FP)(TP + FN)(TN + FP)(TN + FN)}}$
Precision	$\frac{TP}{TP+FP}$
Recall	$\frac{TP}{TP+FN}$

## Supplementary Information

Supplementary Information.

## Data Availability

The weights and network architecture of CNN model trained using the combined learning approach is publicly available at https://github.com/nprksh/landslide-mapping-with-cnn. The repository also contains a Jupyter Notebook, which explains the steps required for mapping landslides in a new area of interest. The inputs required are a DSM and a pair of pre- and post-event Sentinel-2 images. This Notebook can be further forked/modified to map landslides in any other area of interest or with a set of true-color composites from a different sensor. In the same repository, we also deliver four new event-landslide inventories (study areas B, C, E, and F), which were created as a part of this study. The datasets can be used in future studies for the analysis of specific events, as well as in the development of new machine learning methods.
