# Author Correction: A new strategy to map landslides with a generalized convolutional neural network

**DOI:** 10.1038/s41598-022-14634-8

**Published:** 2022-06-16

**Authors:** Nikhil Prakash, Andrea Manconi, Simon Loew

**Affiliations:** Engineering Geology, Department of Earth Sciences, ETH Zurich, 8092 Zurich, Switzerland

Correction to: *Scientific Reports* 10.1038/s41598-021-89015-8, published online 06 May 2021

The original version of this Article contained an error in Table 1, where the formula given for the MCC score was incorrect. The correct and incorrect values appear below.

Incorrect:

**Table:** 

Metric	Formula
F1-Score	$\frac{2TP}{2TP+FP+FN}$
MCC	$\frac{TP\times TN-FP \times FN}{\sqrt{(TP\times FP)(TP\times FN)(TN\times FP)(TN\times FN)}}$
Precision	$\frac{TP}{TP+FP}$
Recall	$\frac{TP}{TP+FN}$

Correct:

**Table:** 

Metric	Formula
F1-Score	$\frac{2TP}{2TP+FP+FN}$
MCC	$\frac{TP\times TN-FP \times FN}{\sqrt{(TP+FP)(TP+FN)(TN+FP)(TN+FN)}}$
Precision	$\frac{TP}{TP+FP}$
Recall	$\frac{TP}{TP+FN}$

The original Article has been corrected.

