# Supplementary material for: A new strategy to map landslides with a generalized convolutional neural network
Source: Sci Rep. 2021 May 6;11:9722. doi: 10.1038/s41598-021-89015-8 (PMC8102623; doi:10.1038/s41598-021-89015-8)
Supplement: Supplementary file 1 — Supplementary Information. [file 41598_2021_89015_MOESM1_ESM.pdf]

A New Strategy to Map Landslides with a Generalized Convolutional Neural Network

Nikhil Prakash[1,*], **Andrea Manconi**[1], **and Simon Loew**[1]

[1]Engineering Geology, Department of Earth Sciences, ETH Zurich, 8092 Zurich, Switzerland
[*]nikhil.prakash@erdw.ethz.ch

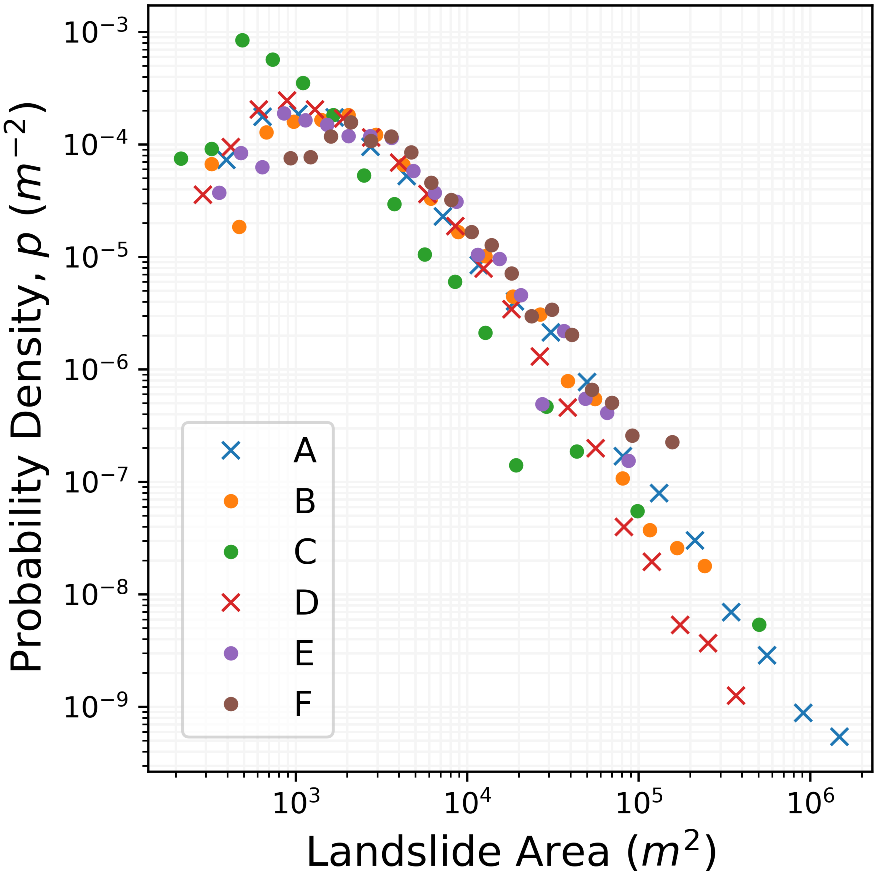

Supplementary Figure 1. The magnitude-frequency plot of the landslides inventories, where $p(A_L)$ is the probability density function, A_L is the area of the landslide, N_{LT} is the total number of landslides in the inventory, and δN_L is the number of landslides with areas between A_L and $A_L + \delta A_L$. The plot with '×' represents inventories taken from already published studies. Conversely, the plot with '·' represents inventories prepared in this study. Study area G had just one large landslide and is therefore not represented in this plot.

The dimension of this images is too big for this page. Please see the full version at: https://github.com/nprksh/landslide-mapping-with-cnn/blob/main/Supplementary-Figure/Supplementary_Figure_2.png.

Supplementary Figure 2. The architecture of the U-Net model used in this study.
